# Association between actual weight status, perceived weight and depressive, anxious symptoms in Chinese adolescents: a cross-sectional study

**DOI:** 10.1186/1471-2458-10-594

**Published:** 2010-10-08

**Authors:** Jie Tang, Yizhen Yu, Yukai Du, Ying Ma, Huiping Zhu, Zhuoya Liu

**Affiliations:** 1 Department of Child, Adolescence & Woman Health Care, School of Public Health, Tongji Medical College, Huazhong University of Science & Technology, 13rd Hongkong Road, Wuhan, PR China

## Abstract

**Backgroud:**

The purpose of this study was to describe actual measured weight and perceived weight and to explore associations with depressive, anxiety symptoms in school adolescents in China.

**Methods:**

A sample of 1144 Chinese adolescents was randomly selected from four schools in Wuhan, China, including 665 boys and 479 girls with ages ranging between 10 and 17 years. Actual measured weight and height and perceived weight status were compared to anxiety and depressive symptoms measured using the revised Self-Rating Anxiety Scale and Children's Depression Inventory. A general linear model was used to compare differences in psychological symptoms among the teenagers with different measured and perceived weights.

**Results:**

When compared with standardized weight tables (WHO age- and gender-specific body mass index (BMI) cutoffs (2007 reference)), girls were more likely to misperceive themselves as overweight, whereas more boys misclassified their weight status as underweight. The adolescents who perceived themselves as overweight were more likely to experience depressive and anxiety symptoms (except girls) than those who perceived themselves as normal and/or underweight. However, no significant association was found between depressive and anxiety symptoms actual measured weight status.

**Conclusions:**

Perceived weight status, but not the actual weight status, was associated with psychological symptoms.

## Background

Adolescence is a critical period when dramatic physical, psychological and social developments take place [1]. With the growth spurt that occurs at this time, most adolescents are very concerned about their body weights and shape, which have been defined as 'body image' [2,3], and how others perceive their body image. Perceived weight is one of the important dimensions of body image and reflects the subjective expectancy of a person's body image [4]. It is reported that adolescents in western societies desire a body image that conforms to the "ideal", a thin shape for women and a lean, muscular shape for men [5,6]. In China, the rapid economic development in the last decade has resulted in accelerated shifts in Chinese dietary patterns and this has led to the significant increase in body mass index (BMI) and a greater risk of overweight and obesity in Chinese populations [7,8]. Dramatic changes also seem to have occurred in cultural beliefs and beauty norms. The notion that a somewhat fat body mass is a symbol of family wealth is now changing in the cities with the influence of the "ideal beauty norm" from the western societies. The pursuit of "ideal" slim body image has become a social phenomenon among female Chinese adolescents in large cities, especially the high school and college students [9-11].

The "ideal" body images are usually affected by what appears in the mass media, other cultural outlets, and the attitudes of their peers. Previous studies in the west posited that pressures to be thin from family, peers, and media contribute to body dissatisfaction [12]. Todd indicated that appearance-related social pressure and social comparison as well as appearance concerns did not directly reflecting body size or weight [13]. Recurring messages from the media, family, and peers that one is not thin enough, affect the "ideal" body image. These pressures foster discontent with the individual's body size or body weight.

Excessive pursuit of the "ideal" body image reflects indirectly the dissatisfaction with one's body image, which consists of body image or body shape dissatisfaction and body weight dissatisfaction [14]. Misperception of weight status is also a kind of indirect body weight dissatisfaction. Research on body weight dissatisfaction has shown that adolescents are often split between those who desire to lose weight and those who desire to gain weight [15]. Thus body weight dissatisfaction is associated with either perceived underweight or overweight.

The consequences of obesity are far reaching and extend into physical and psychosocial conditions [16]. However, unlike physical effects from obesity, the negative psychological effects of body weight dissatisfaction are inconsistently reported [17-19]. In the US, it has been reported that overweight youths have increased morbidity and are at risk of negative psychosocial outcomes, such as social stigmatization, suicidal ideation, and low self-esteem [20]. Studies conducted in China also suggest that overweight or obese students have experienced more emotional problems and lower social cooperation skills than normal weight students [21]. However, Goodman and Whitaker noted that obesity did not predict depression [22]. A recently study conducted in Germany also showed that adolescents who misperceived their body weight were more likely to accept interventions to lose weight, compared to those who were actually overweight or obese [23]^.^

Why do these discrepancies occur? Some studies pointed that there was little question that weight in itself was important to adolescents, while the real problem of excess adiposity, with an equal importance, was the adolescent's perception of what defines normal weight [24]. The conception of "I am fat" may account for the negative psychological symptoms among adolescents, and this hypothesis has been supported in previous studies conducted in the west. While the perception of weight can be influenced by many social factors, little is known about the association between body weight, weight concerns and psychological symptoms in the Chinese culture.

The aims of this study were to describe the actual weights, and perceived weights of Chinese adolescents and to evaluate their association with symptoms of depression and anxiety. We hypothesized that individuals who have a discrepancy between perceived weight and actual weight would be more likely to report symptoms of anxiety and depression.

## Methods

### Study Population

This study used a stratified multistage sampling. First, one urban and one rural area where the socioeconomic status was representative of the general level of urban and rural areas, respectively, were selected. Then, in each selected area, one junior high school and one high school were selected randomly. Finally, in each of the selected schools, two classes were randomly selected for each grade. All the students in the selected classes were eligible for inclusion in the study. For the final sample 1231 pupils were selected, with 36 students absent from the school at the time of survey. The questionnaire was administered to 1195 students, and no subjects declined to participate in the study. The inclusion criteria for age in the present study ranged from 10 to 17 years. In the final sample 32 students were excluded due to incomplete questionnaires and 19 additional students were excluded due to being older than 17 years. Thus, the final study population consisted of 1144 students with a mean age of 13.9 (SD = 1.9), and the effective response rate was 92.7%. The study included 665 boys and 479 girls, and an informed consent was obtained from each of students and their parents. The Ethical Committee of the Medical Association of Tongji Medical College of Huazhong University of Science and technology approved the study.

Data collection was carried out in March 2007 by a group of trained investigators. The investigators explained the purpose and procedures of the study and provided assistance in filling out questionnaire on the spot. Before answering the questionnaire, the students were told that the questionnaire did not represent a test and that there were no correct or incorrect answers. The same announcements were printed on the first page of the questionnaire. Emphasis was placed on answering the self-rated questionnaire alone, honestly and accurately.

### Measurement of Body Weight and Height

Height and body weight were measured by a medical examiner. The height was measured to the nearest 0.5 cm using a stadiometer. Body weight was rounded to the nearest 0.1 kg on a standard physician's beam scale, with the subjects dressed in light underwear and no shoes. The body mass index (BMI) was calculated as body mass divided by height squared (kg/m^2^). Based on the international norms from the WHO (2007 reference) with age (to the nearest one month) and gender-specific BMI [25], BMI cutoffs were the following: overweight, BMI >1SD; obesity, BMI >2SD; thinness, BMI < -2SD; and severe thinness, BMI < -3SD.

### Perceived Weight Status

Each student was asked by "Which body shape do you think you have?" to describe their body weight status with five response options: too thin; relatively thin; all right; relatively heavy; and too heavy. Self-perception of body weight status was then correlated with the measured BMI. Students who described themselves as relatively thin or very thin, but were not underweight based on their measured BMI were classified as "misconception of underweight." Similarly, subjects who described themselves as relatively heavy or too heavy but were not overweight based on their BMI were classified as "misconception of overweight." Subjects who correctly estimated their body weight status were classified as "correctly estimated."

### Pubertal Status

Pubertal status was assessed using different characteristics for each sex. Assessed characteristics included breast development and/or menarche in girls, and voice change and/or facial hair growth in boys [11]. The original questions were: "How old were you when your menarche and/or breast development happen?" (For girls), and "How old were you when your voice change and/or your beard appear?" (For boys). Subjects reported "not happened yet" were defined as "not entered puberty", subjects reported with a specific age were defined as "having entered puberty".

### Depressive Symptoms

The Children's Depression Inventory (CDI) developed by Kovacs. is a self-rating scale widely used to assess depressive symptomatology in children and adolescents age 8 to 17 years. It consists of 27 items each with scores ranging from 0 to 2, and yields total scores from 0 to 54. The higher scores reflecting greater symptomatology. A score of 19 is the criterion score for identifying symptoms consistent with clinical depression. The Chinese version of the CDI has been validated in Chinese children [26]. Cronbach's α of the CDI measure in the Chinese sample was 0.85 [27].

### Anxiety Symptoms

The revised Self-Rating Anxiety Scale (Zung SAS) was used to evaluate the level of anxiety related symptoms during the last week before the survey [28]. This self-administered test has 20 questions, with 15 items worded toward increasing anxiety levels and five questions worded toward decreasing anxiety levels. Each question was scored on a scale of 1-4 (rarely, sometimes, frequently, and always). The scores ranged between 20 and 80. Higher scores reflected more severe anxiety. The internal consistency as reflected by Cronbach coefficient alpha of these twenty items in our study sample was 0.756.

### Statistical Analysis

Descriptive statistics (mean, standard deviation, and percentage) were calculated to reflect the background characteristics of the sample. Chi-square tests and *t*-tests were used to compare frequencies and continuous data, respectively. A general linear model was generated to compare psychological differences among different actual and perceived weight groups with adjustment for age and regions. All models were stratified by gender. Significance level was set at 0.05. Statistical analyses were conducted using Statistical Package for Social Sciences software (SPSS for Windows 15.0, SPSS Inc., Chicago, IL).

## Results

General characteristics of the boys and girls are summarized in Table 1. A total of 1144 students (665 boys and 479 girls) from the four selected schools were included in this study, and 79.5% of the sample (72.8% of boys and 88.9% of girls) had entered puberty. Boys were significantly heavier and taller than girls in our survey (*p *< 0.01). The boys in rural area were significantly taller but thinner than boys in the urban area (*p *< 0.05), and girls from the rural area were significantly higher than girls in the urban area (*p *< 0.05). However, a comparison of weight between girls in rural and urban areas was not statistically significant. There was no significant difference in mean body mass index (BMI) between boys and girls, nor was there any significant difference in overweight and underweight rate between boys and girls (overweight: 13.2% vs. 11.5%, χ^2 ^= 0.78, *p *= 0.38; underweight: 3.6% vs. 2.5%, χ^2 ^= 1.11, *p *= 0.29). The difference in overweight and underweight prevalence between the subjects living in the urban area and those living in the rural area was statistically significant (22.8% for urban vs. 6% for rural, χ^2 ^= 70.11, *p *< 0.001), whereas underweight rate was significantly lower in urban than that in rural areas (0.9% vs. 4.6%, χ^2 ^= 11.94, *p *= 0.001).

**Table 1:** Descriptive characteristics of the study subjects

	Boys (n = 665)	Girls (n = 479)	All (n = 1144)
Age (Mean ± SD)	14.5 ± 1.8	14.4 ± 1.9	14.4 ± 1.8
Residence (%)			
Urban	39.9	37.9	38.7
Rural	60.1	62.1	61.3
Weight (kg, Mean ± SD)	51.3 ± 10.7	48.6 ± 9.3	50.2 ± 10.2
Height (cm, Mean ± SD)	161.4 ± 9.2	157.3 ± 6.9	159.7 ± 8.6
BMI (kg/m^2^, Mean ± SD)	19.5 ± 2.9	19.5 ± 3.1	19.5 ± 3.0
Weight status (%)*			
Overweight	13.2	11.5	12.5
Urban	27.4	16.8	22.7
Rural	4.6	8.0	6.0
Normal	83.2	86.0	84.4
Urban	71.8	82.2	76.4
Rural	90.1	88.5	89.4
Underweight	3.6	2.5	3.1
Urban	0.8	1.0	0.9
Rural	5.3	3.5	4.6
Perceived weight status (%)			
Too thin	7.2	2.7	5.3
Urban	4.4	3.1	3.8
Rural	9.0	2.4	6.3
Relative thin	34.1	17.3	27.1
Urban	31.3	18.8	27.8
Rural	35.8	16.3	26.0
All right	44.2	55.1	48.8
Urban	43.7	47.1	51.1
Rural	44.6	60.4	45.1
Relative heavy	12.9	23.6	17.4
Urban	17.5	29.8	14.0
Rural	10.2	19.4	22.8
Too heavy	1.5	1.3	1.4
Urban	3.2	1.0	0.9
Rural	0.5	1.4	2.3

*: Overweight and underweight both were defined according to the norm (2007) provided by WHO. Overweight: age- and sex-specific over Mean + SD; underweight: age- and sex-specific over Mean - 2SD. More details please link to http://www.who.int/growthref/who2007_bmi_for_age/en/index.html

### The effect of demographic features of perceived weight and body satisfaction

Boys were more likely than girls to perceive themselves as 'too thin' (7.2% vs. 2.7%, χ^2 ^= 11.2, *p *= 0.001) or 'relatively thin' (34.1% vs. 17.3%, χ^2 ^= 39.8, *p *< 0.001). However, girls were more likely than boys to consider themselves as 'relative heavy' (23.6% vs. 12.9%, χ^2 ^= 22.1, *p *< 0.001). There was no difference between genders in the perceived weight status of being too heavy (1.5% vs. 1.3%, χ^2 ^= 0.127, *p *= 0.72). Urban teenagers were more likely than rural teenagers to regard themselves as being 'relatively heavy' (22.8% vs. 14.0%, χ^2 ^= 14.7, *p *< 0.001) or ''too heavy (2.3% vs. 0.9%, χ^2 ^= 3.87, *p *= 0.049). In addition, 3.8% of urban teenagers and 6.3% of rural teenagers classified themselves as 'too thin', and 26% teenagers in urban and 24.3% teenagers in rural areas considered themselves as 'relatively heavy' (*p *> 0.05).

### The relationship between actual weight and perceived weight

The frequencies of actual and perceived weight status are listed in Table 2. Comparing the perceived weight status to actual weight status, 48.1% of subjects misclassified their body weight with 30.6% misclassifying themselves as underweight, 5.5% misclassifying themselves as normal weight, and 12.0% misclassifying themselves as overweight. Further, 41.8% of girls and 52.6% of boys misclassified their body weight (χ^2 ^= 13.2, *p *= 0.000), whereas 17.7% of girls compared with 7.8% of boys who were actually normal or underweight misclassified themselves as either relatively heavy or too heavy, (χ^2 ^= 26.1, *p *< 0.001). However, boys who were actually normal or overweight were more likely than girls to describe themselves as underweight (39.1% vs. 18.8%, χ^2 ^= 54.1, *p *< 0.001). There was no significant difference in misconception of overweight between the urban teenagers and the rural teenagers (27.1% for urban vs. 31.5% for rural, χ^2 ^= 2.56, *p *= 0.11) and underweight (10.4% for urban vs.13.0% for rural, χ^2 ^= 1.74, *p *= 0.19).

**Table 2:** Frequency distribution of actual and perceived weight status

	Perceived weight status			
	
	Underweight	Normal weight	Overweight	Total
Actual underweight (n, %)				
Boys				
Urban	1(50.0)	0(0)	1(50.0)	2(100)
Rural	14(63.6)	8(36.4)	0(0)	22(100)
Girls				
Urban	2(100)	0(0)	0(0)	2(100)
Rural	4(40.0)	6(60.0)	0(0)	10(100)
Actual normal weight (n, %)				
Boys				
Urban	80(44.2)	88(48.6)	13(7.2)	181(100)
Rural	166(44.6)	168(45.2)	38(10.2)	372(100)
Girls				
Urban	40(25.5)	85(54.1)	32(20.4)	157(100)
Rural	48(18.8)	154(60.4)	53(20.8)	255(100)
Actual overweight (n, %)				
Boys				
Urban	9(13.0)	22(31.9)	38(55.1)	69(100)
Rural	5(26.3)	8(42.1)	6(31.6)	19(100)
Girls				
Urban	0(0)	5(15.6)	27(84.4)	32(100)
Rural	2(8.7)	14(60.9)	7(30.4)	23(100)
Total	371	558	215	1144

### The effect of perceived weight on depressive and anxious symptoms

All the data were stratified by gender and were used to compare symptoms of depression and anxiety among the different categories of perceived weight and adjusted for age and region (rural and urban areas). Results are summarized in Table 3. Significant differences were found in depressive symptoms for boys and girls, and anxious symptoms for boys. Boys who describe themselves as overweight were more likely to experience higher levels of anxiety and depressive symptoms than boys with either perceived underweight or normal weight boys (*F *= 4.21, *p *= 0.02; *F *= 3.93, *p *= 0.02). Similarly, girls who perceived themselves as overweight experienced significantly higher levels of depressive symptoms than those who perceived themselves as underweight or normal (*F *= 5.07, *p *= 0.01). However, the difference in anxiety symptoms for girls among these groups was not statistically significant (*F *= 2.13, *p *= 0.12). Both in boys and girls, there was no significant differences in depressive or anxiety symptoms between subjects who misperceived themselves to be overweight and those who correctly described their overweight status (*p *= 0.089, *p *= 0.123). Similarly there were no differences when comparisons were made between subjects who misperceived themselves to be normal or underweight and those who correctly describe their weight status (normal or underweight).

**Table 3:** Comparison of depressive and anxiety symptoms among different categories of perceived weight in boys and girls adjusted for age and areas

	Boys				Girls			
		
	Mean	SD	F	*p*	Mean	SD	F	*p*
Depressive symptoms								
Perceived underweight	10.1	7.5			10.0	7.0		
Perceived normal	9.5	7.5	4.21	0.02	9.4	8.2	5.07	0.01
Perceived overweight	11.9	8.0			12.0	7.3		
Anxiety symptoms								
Perceived underweight	30.7	6.3			3.17	5.8		
Perceived normal	31.9	7.6	3.93	0.02	32.4	6.6	2.13	0.12
Perceived overweight	32.6	6.0			33.7	6.6		

### The effect of actual measured weight on symptoms of depression and anxiety

A general linear model was also fitted to find any differences in depressive and anxiety symptoms among the different kinds of actual weight status and adjusted for age and region (rural and urban), Results are summarized in Table 4. No significant differences were found in depressive symptoms for boys and girls (*F *= 2.45, *p *= 0.09; *F *= 1.74, *p *= 0.18). Similarly, the difference in anxious symptoms for boys and girls among these groups were also not statistically significant (*F *= 1.89, *p *= 0.15; *F *= 1.43, *p *= 0.24).

**Table 4:** Comparison of depressive and anxiety symptoms among different categories of actual weight in boys and girls adjusted for age and areas

	Boys				Girls			
		
	Mean	SD	F	*p*	Mean	SD	F	*p*
Depressive symptoms								
underweight	9.7	7.7			9.2	7.4		
normal	10.0	7.5	2.45	0.09	10.3	7.8	1.74	0.18
overweight	13.0	9.1			11.6	7.5		
Anxiety symptoms								
underweight	31.1	6.9			29.0	5.1		
normal	31.0	6.9	1.89	0.15	32.7	6.5	1.43	0.24
overweight	32.3	6.9			32.6	6.1		

### Subgroup analysis

Weight perception and its relationship with depressive and anxious symptoms may be effected by the developmental phases of the teenagers and hence, stratified analyses were conducted for "pre-pubertal" and "pubertal" samples separately to compare their perceived weight status. In the pre-pubertal group, no significant gender difference was found in weight perception (χ^2 ^= 2.22, *p *= 0.33), whereas in the pubertal group, boys were more likely than girls to perceive themselves as relatively thin or too thin (39.3% vs. 15.3%, χ^2 ^= 68.4, *p *< 0.001), and girls were more likely than boys to considered themselves as too heavy (26.5% vs. 14.9%, χ^2 ^= 68.4, *p *< 0.001). Comparison of perceived weight status between boys and girls stratified by age showed no significance between boys and girls at the age of 10 and 11, However, significant difference was found between boys and girls at the age of 13, 14, 15, 16 and 17, and the results were similar to prepubertal vs. pubertal comparisons (because most prepubertal teenagers were in the age range of 10 to 11). In pubertal teenagers who misperceived their weight status, girls were more likely to misperceived themselves as overweight than boys (73.8% vs. 37.3%, χ^2 ^= 56.7, *p *< 0.001); similarly, boys tended to misperceived themselves as underweight (48.5% vs. 17.2%, χ^2 ^= 56.7, *p *< 0.001). No gender difference in misclassification of weight status was found for pre-pubertal subjects. Significant effects on depressive symptoms were only found in pubertal girls (*F *= 4.71, *p *= 0.009) and in pre-pubertal boys (*F *= 6.95, *p *= 0.001). In both boys and girls stratified by pubertal status, there were no significant differences in depressive and anxious symptoms among subjects who misperceived themselves to be overweight, underweight, and those who correctly described their overweight status.

## Discussion

Our study revealed that more boys than girls thought of themselves as underweight, whereas more girls considered themselves to be overweight. The results were consistent with previous findings on adolescents' self-perception of body weight in China[10,29].

Perceived weight, as one of the important aspects of body image, indirectly reflects subjective expectancy of body weight or "ideal" body weight [4]. It may be affected by the predominant culture, physique of peers, media and by how others perceive their body weight and shape. Being thin is desired in Western societies, and many normal weight adolescents, especially girls, perceive themselves as being overweight and try to lose weight to achieve the socially endorsed ideal of a beautiful body [30-32]. In China, in the past two decades after the Chinese government adopted the economic reform policy, larger cities in the mainland, such as Wuhan in the present study, have been considered to be the most economically developed and modernized regions. Adolescents and youth in these areas have not only been experiencing a fast-growing economy but have also been exposed to pervasive imported western culture. Beliefs to guide action were once attributed to having a plump physique, a somewhat fat body mass was considered to be a symbol of family wealth. While this belief may still exist in some rural, economically less developed areas, it has become a history in urban areas and is gradually being replaced by the preference for a slim physique in adolescents and youths, especially for girls, even if this "slim physique" may be unhealthy or unrealistic [10,33]. A survey of abnormal eating behavior among female students in middle school in Beijing showed that the actual BMIs of the subjects were in the normal range, but the subjects' ideal BMI was lower than the normal standard [34]. In our study, 24.8% of the girls regarded themselves as too heavy or relatively heavy. Among this group of teenaged girls, 71.4% of them had a weight in the normal range, whereas 41.3% of the boys describe themselves as relatively thin or too thin, but 89.5% actually had normal weight and 5.1% were overweight. The actual underweight boys only accounted for 5.5% in our study group and 94.6% of boys who considered themselves as underweight misperceived their actually weight status. More boys than girls in our study misperceived their body weight (52.3% vs. 41.8%), and this may be because girls were more concerned about their body weight than boys, and thus more often to measure their body weight.

Body weight dissatisfaction and fear of fatness in early adolescence are important risk factors for eating disorders [14] and are considered to be significant health concerns among health professionals worldwide [35]. Surveys conducted in the USA and Caribbean showed that youths reported higher levels of extreme dieting behaviors such as vomiting, taking diet pills and diuretics [21,36]. In China, there were fewer reports on controlling weight with extreme behaviors; however, dieting blindly is widespread [34]. Notably, evidence suggests that the risk of engaging in these behaviors is higher among people with certain psychiatric conditions, particularly bulimia nervosa and anorexia nervosa [37].

According to social psychology theories regarding negative self-appraisal and self-rejection, it is possible that failure to meet socio-cultural norms of body shape and weight is related to levels of depressive symptoms [38]. However, whether overweight and obesity are correlated with depressive and/or anxious symptoms is unclear. In the present study, we found that negative body image expressed by perceived overweight or underweight were associated with depressive and/or anxious symptoms; however, the association between actual weight status and psychological symptoms for teenagers was not statistically significant. Inconsistent with our results, Tao et al [39] reported that the rates of depressive and anxious symptoms were 37.1% and 22.9% in obese group, 14.5% and 6.5% in overweight group, 22.0% and 7.0% in control group respectively. Similar with our results, Friedman and Judi reported that no association was found between depressive symptoms and body mass index. However, perception of weight related to higher depressive symptoms [24,40]. The difference between our study and Judi's still existed, because our results showed that teenagers who perceived themselves as overweight experienced more serious depressive symptoms than those teenagers who perceived themselves as normal/underweight. The "dose response relation" seemed to exist between perceived weight and depressive symptoms. This association was not found between perceived weight and anxiety symptoms. However Judi did not report the "dose response relation" between the two variables, and gave a cautious conclusion after reviewing the single item measure of depressive symptoms.

In addition, an association between body image and depressive symptoms is also supported by the findings from neurobiological investigations. Potentially common neurobiological abnormalities involved in disorders of mood and weight regulation include deficits in the serotonin system and the hypothalamic-pituitary-adrenal axis, as well as deficits in the brain circuits that underlie hedonic regulation [40]. In spite of this, a general comparison between actual weight and perceived weight and psychological symptoms in our study may not be the best approach, because perceived body weight based on actual weight and affected by other factors, thus, our results seemed to suggest that body image or weight perception may provide a link between actual body weight and psychological symptoms [41].

Finally, in our results there were no significant gender differences observed in either depressive or anxious symptoms with different weight perceptions. Boys and girls who perceived themselves to be overweight showed higher scores than those who perceived themselves as normal or underweight. These results differ from some previous studies in Western countries as Cortese reported that the association between body size and depressive symptoms in young adolescents was curvilinear and was moderated by sex [42]. The differences between these two studies may be due to the effects of culture disparity. Boys' pursuit of strength were obviously a trend in our survey, because 41.3% of boys described themselves as too thin or relative thin and 94.5% of those who regarded themselves as underweight misclassified their weight status.

The present study has some limitations. First it is possible that overweight or obese teenagers may be absent from school, but the number was relatively small. Nonetheless, the mean BMI of the total sample (males and females) was similar to those found in another independent study conducted on a sample of children of the same age range and living in the same geographical area, and thus, it is likely that the BMI distribution of our sample was representative of that of the entire population of Wuhan, China [43]. Second, we did not consider social economic status (SES), which may effect the pathway of the observed associations between weight perception and psychological factors. In our study, we designed some questions to estimate SES, however, the responses were either missing or non-informative (up to 85% of the data). This is mainly due to the fact that most of teenagers did not know the SES of their family. Third, not including measures of body image and self-esteem at this survey may fail to explore the pathway of the observed associations between weight perception and psychological factors. Finally, the nature of cross-sectional study design cannot investigate the direction of causality.

## Conclusion

This work shows that it is perceived weight status but not the actual weight status that is associated with psychological symptoms. Hence, distortion of weight perception may have a detrimental influences on psychological development of adolescents, and this results may useful for programs aimed at preventing mental problems in early adolescence.

## Competing interests

The authors declare that they have no competing interests.

## Authors' contributions

JT and YY participated in the design of the study and performed the statistical analysis. HZ drafted the manuscript, YM and ZL participated in data collection. YD participated in its design and coordination and helped to draft the manuscript. All authors read and approved the final manuscript.

## Pre-publication history

The pre-publication history for this paper can be accessed here:

http://www.biomedcentral.com/1471-2458/10/594/prepub

## Ackowledgements

The authors acknowledge the support of grants from the National High-Tech Research and Development Program ("863" Program) (Grant No 2006AA02Z427). Opinions reflect those of the author and not necessarily those of the granting agencies. The authors also express their gratitude to Professor (tenured) Qingyi Wei, Department of Epidemiology, Division of Cancer Prevention and Population Sciences, The University of Texas M. D. Anderson Cancer Center, Houston, TX, for his efforts of editing and revision.
